# A Positive Association between Working Memory Capacity and Human Creativity: A Meta-Analytic Evidence

**DOI:** 10.3390/jintelligence11010015

**Published:** 2023-01-13

**Authors:** Zheng Gong, Kuan Miao, Xuerong Liu, Mengjie Luo, Yang Yu, Zhiyi Chen

**Affiliations:** 1 School of Psychology, Army Medical University, Chongqing 400038, China; 2 Experimental Research Center for Medical and Psychological Science (ERC-MPS), Army Medical University, Chongqing 400038, China; 3 College of Basic Medicine, Army Medical University, Chongqing 400038, China

**Keywords:** creativity, working memory, meta-analysis, cultural environment, working memory capacity

## Abstract

Creativity serves as a fountain for social and scientific development. As one of the most crucial human capabilities, creativity has been believed to be supported by the core component of higher cognitive functions—working memory capacity (WMC). However, the evidence supporting the association between WMC and creativity remains contradictory. Here, we conducted a meta-analysis using random-effects models to investigate the linear association between WMC and creativity by pooling the individual effect size from the previous literature. Further, a subgroup analysis was performed to examine whether such association is specific for different WMC categories (i.e., verbal WMC, visual–spatial WMC and dual-task WMC). The main meta-analytic results showed a significantly positive association between WMC and creativity (*r* = .083, 95% CI: .050–.115, *p* < .001, n = 3104, k = 28). The subgroup analysis demonstrated consistent results by showing a significantly positive association between them, irrespective of WMC category. We also found that cultural environments could moderate this association, and we identified a strong correlation in participants from an Asian cultural context. In conclusion, this study provides the evidence to clarify the positive association between WMC and creativity, and implies that the Asian cultural context may boost such an association.

## 1. Introduction

Creativity refers to the ability to produce novel and suitable ideas in a specific environment (Sternberg and Lubart 1999). Based on Guilford’s divergent thinking test, it is defined as the composite concepts of originality, flexibility, and novelty of thinking (Guilford 1968). Creativity facilitates the generation of ideas in a problem-solving context and drives scientific discoveries and human progress (DeHaan 2009). Creativity was also found to be a phenotype associated with mental health problems, such as anxiety (Reid et al. 1959), schizophrenia (Degmečić 2018), and children’s behavioral problems (Fancourt and Steptoe 2019). As one of the most crucial human-specific capabilities, creativity has been intensively studied to uncover what “cognitive cornerstones” are, with working memory being a research hot spot (Hennessey and Amabile 2010; Ovando-Tellez et al. 2022).

Growing evidence suggests that executive functions (EFs) play an important role in creativity (Zabelina et al. 2019). However, it remains unclear which EF-specific components are involved. EF refers to a series of high-order cognitive functions that are essential to ensuring physical and mental health, as well as academic and career success; EF contains three core components: inhibition, working memory, and cognitive flexibility (Diamond 2013). Among them, working memory (WM) refers to the capability to hold and manipulate information temporarily with “block-wise entities” (Baddeley 2012). To structurally quantify WM ability, working memory capacity (WMC) was broadly adopted for encapsulating information in both storage and processing stages (Wagner et al. 2021). With a fundamental role in cognition, WMC is found to be indispensable for knowledge acquisition, complicated reasoning, problem-solving, and so on (Cowan 2014; Miller et al. 2018; Wiley and Jarosz 2012). Moreover, WM impairment is a hallmark of many mental illnesses, such as anxiety (Cowan 2014), Attention Deficit/Hyperactivity Disorder (ADHD) (Alderson et al. 2013), and schizophrenia (Gold et al. 2018).

It has long been acknowledged that creativity is one of the most crucial factors associated with the development of multifarious cognitive components (De Dreu et al. 2012; Dygert and Jarosz 2020). However, the specific role of WMC in creativity remains to be clarified. The dual-pathway to creativity model suggests that WMC can reflect cognitive flexibility and cognitive persistence to positively predict creativity (Baas et al. 2008). A certain number of studies provided empirical evidence to support the positive role of WMC in creativity. For instance, Teng et al. (2018) demonstrated that increasing WMC prominently improved information extraction efficiency in creative activities (Teng et al. 2018). Further, Vally et al. (2019) demonstrated that almost all the domains in creativity ability (i.e., originality, elaboration, and fluency) could be improved by a 13-week WMC training (Vally et al. 2019). In addition, indirect evidence also supports the association between WMC and creativity: individuals with high WMC were found to outperform in creative tasks, insight-problem-solving and creative thinking (De Dreu et al. 2011; Korovkin et al. 2018; Murray and Byrne 2013; Orth et al. 2019). As for brain-behavior association, brain functional or neuroanatomical changes supporting WMC (e.g., increased functional connectivity of frontoparietal network) may be in favor of creativity ability (Chen et al. 2018; Sun et al. 2016; Zhuang et al. 2021). In summary, several lines of evidence support that WMC could be a fundamental factor promoting creativity.

In contrast to the above results, another theory, known as the controlled attention theory, posits that individuals with a higher WMC are more easily confined within a single domain, which is detrimental to the implementation of creativity (Beaty and Silvia 2012). Related research findings have raised questions regarding the association between WMC and creativity. Fugate and colleagues (2013) reported the significantly poorer performance of children with a high WMC in creative tasks, compared to children with a low WMC (Fugate et al. 2013). In addition, Furley et al. have extended a similar conclusion into adults, by showing a negative association between WMC and creativity in adult athletes (Furley and Memmert 2015).

To make things more complicated, a portion of studies argued that WMC does not show any impact on creativity, by finding that WMC is not a robust predictor for creativity ability (Chein and Weisberg 2014; Gilhooly and Fioratou 2009). Theoretical evidence underscored the null association between WMC and creativity also: creative problem solving was theoretically defined as a non-conscious process that does not appear to link WM/concentration with creative activities (Wang et al. 2020; Xinru Zhang et al. 2019). Despite evidence for the positive association between WMC and creativity, these conflicting findings challenge such arguments.

To address these conflicting results, meta-analysis has been widely used as a potent tool by providing evidence, based on the extensive previous literature (Egger and Smith 1997; Gajda et al. 2017; Peng et al. 2018). By synthesizing prior evidence into meta-analytic models, the “true effect” could be examined to confront contradictory independent observation, that is, the current meta-analysis may detect the “true effect” of the association between WMC and creativity (Michael Borenstein 2022; Brockwell and Gordon 2001).

Therefore, the current study aimed to provide evidence to clarify the association between WMC and creativity. We conducted a meta-analysis with a random-effects model to pool the individual effect size from each study concerning the association between WMC and creativity. The systematic retrieval of the literature was conducted by following 2020 PRISMA pipeline in PsycINFO, Web of Science, PubMed, EMBASE, CNKI (Chinese database) and PsycARTICLES datasets on 17 June 2022. Further, to probe into the potential hierarchical factors affecting this association, we conducted an exploratory, subgroup meta-analysis by dividing comprehensive WMC into verbal WMC, visual–spatial WMC and dual-task coordination WMC. Finally, to further probe into the impact of potential confounding factors for the meta-analytic effects, we conducted a moderation analysis to examine whether the association between WMC and creativity is moderated by cultural background and age group.

## 2. Materials and Methods

**Figure 1:**
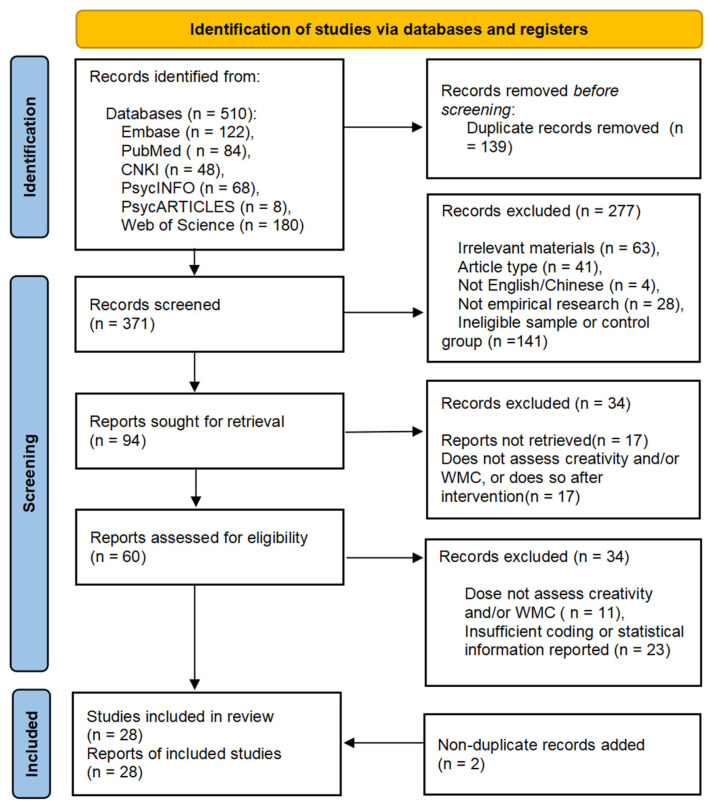
Flow diagram of study selection process based on the 2020 PRISMA protocol.

To improve reproducibility and transparency as recommended, this study adhered to the Preferred Reporting Items for Systematic reviews and Meta-Analyses (PRISMA) and CHARMS pipelines (Page et al. 2021) (see Figure 1). Further, all the materials relating to the present study were deposited at the Open Science Framework (OSF) with open access. This meta-analysis mainly followed five steps: (1) developing a searching strategy for retrieval of the literature; (2) defining the inclusion and exclusion criteria; (3) screening the eligibility of the literature by using the inclusion and exclusion criteria; (4) the targeting data were extracted, coded, and assessed for evidence-based quality; and (5) statistics were estimated for pooling the individual effect size by building meta-analytic and moderation models.

### 2.1. Search Strategy

For completeness and accuracy of the literature search, we used a keyword-based retrieval strategy to search in Boolean logic in PsycINFO, Web of Science, PubMed, EMBASE, CNKI and PsycARTICLES datasets. Specific Boolean expressions were as follows: (“Memory, Short-Term” OR “working memor*” OR “phonological loop” OR “visuospatial sketchpad” OR “central executive” OR “verbal working memory” OR “visuospatial working memory” OR “executive function” OR “updating”) AND (“Creativity” OR “creative activit*” OR “creative thinking” OR “creative achievement*” OR “creative imagination*” OR “creative personalit*”) NOT (review OR meta-analysis). To ensure data pooling completeness, reference lists of included articles, published in the last two years, were hand-reviewed.

### 2.2. Study Selection

According to the research objectives we predefined, the inclusion criteria were defined was as follows: (1) WMC and creativity should be measured by using standardized scales or board-certified behavioral tasks; (2) fundamental statistics (e.g., Pearson’s correlation coefficient, sample size) for examining the association between WMC and creativity should be presented clearly; (3) peer-reviewed journal articles and dissertations are allowed; (4) analytic data should be self-recruited (i.e., independent dataset); (5) a sample or control group would be qualified; (6) creativity and/or WMC would be assessed without intervention; and (7) studies should be in English/Chinese language only. On the other hand, the exclusion criteria were as follows: (1) systematic reviews (with or without meta-analysis) or preprints were not be accepted; (2) non-standardized measures were used to estimate WMC or creativity; and (3) statistics were reported vaguely.

### 2.3. Encoding and Statistical Analysis

Meta-information was extracted from these included studies, including the author’s name, publication date, sample size, age, and sample populations (nations and identity). Further, tasks for measuring WMC were extracted and coded into three domains: verbal WMC task, visual–spatial WMC task and dual WMC task. In addition, measures for quantifying creativity were extracted and coded into the following categories: Torrance Tests of Creative Thinking (TTCT), Test of Creative Thinking–Drawing Production(TCT-DP), Abbreviated Torrance Test for Adults (ATTA), Divergent thinking tests (DT), Convergent thinking tests (CT), Williams Prefer Measurement Forms (WPMF), Williams Creativity Assessment Packet (WCAP), Unusual uses task (UUT), Alternative Uses Task (AUT), Consensual Assessment Technique (CAT), Creative Achievement Questionnaire (CAQ), Associative fluency tasks (AF) and the Remote Association Test (RAT). Finally, for pooling the individual effect size into the meta-analytic model, the statistics (r value) and sample size for each included study were extracted.

### 2.4. Quality Analysis

To ensure the data quality, all the data that were extracted and coded from included studies were cross-validated by two independent researchers (IRs, GZ and MK). Any disagreements of data extraction and coding were solved by the third IR (CZY). Furthermore, two additional assessors (LXR and LMJ) were recruited to evaluate evidence-based quality by using a modified Newcastle–Ottawa Scale (mNOS) (Lo et al. 2014). The mNOS included five items to evaluate the risk of bias (ROB) for evidence (study) quality, with high ROB for total scores of ≤3 for each study. The specific assessment of mNOS included the following: (1) sample representativeness; (2) sample size; (3) comparability between respondents and non-respondents; (4) quantitative study quality; and (5) reporting quality for statistics.

### 2.5. Statistical Analysis

Comprehensive Meta-Analysis Software version 3.0 (CMAV3.0) was used to implement all the data analysis as we mentioned above (Makinde et al. 2021). To determine which statistical model is suitable in the current analysis, between-study heterogeneity, across the included studies, was estimated by using Higgins and Thompson’s I^2^ test (Borenstein et al. 2011) and Cochran’s Q test. As recommended, the random-effects meta-analytic model is suitable to pool individual effect size by controlling high between-study heterogeneity (I^2^ > 50%, *p*-value < 0.1). In addition to this main analysis, the sub-group meta-analysis was deployed to validate the individual meta-analytic effect for this association by three WMC tasks, including verbal, visual–spatial, and dual-task coordination. Furthermore, to examine whether the meta-analytic effect is biased by confounding factors, we built the moderation-effect models by taking the cultural background and age group into account. Finally, for quality control, publication bias was inspected by producing funnel plots and was calculated by using Egger’s test and Kendall’s test (Sterne and Egger 2005).

## 3. Results

Here, a total of twenty-eight papers (k = 28, the number of r statistics = 75, n = 3104) were screened and deemed eligible for generating the final data pool in the following meta-analysis. Fundamental information for all the included studies is tabulated in Table 1.

### 3.1. Main Meta-Analysis

The results of the heterogeneity tests revealed high between-study heterogeneity in this meta-analytic model, by showing a significantly high I^2^ value (I^2^ = 55%, *p* < .001). Thus, the random-effects models were built for the following meta-analysis.

**Figure 2:**
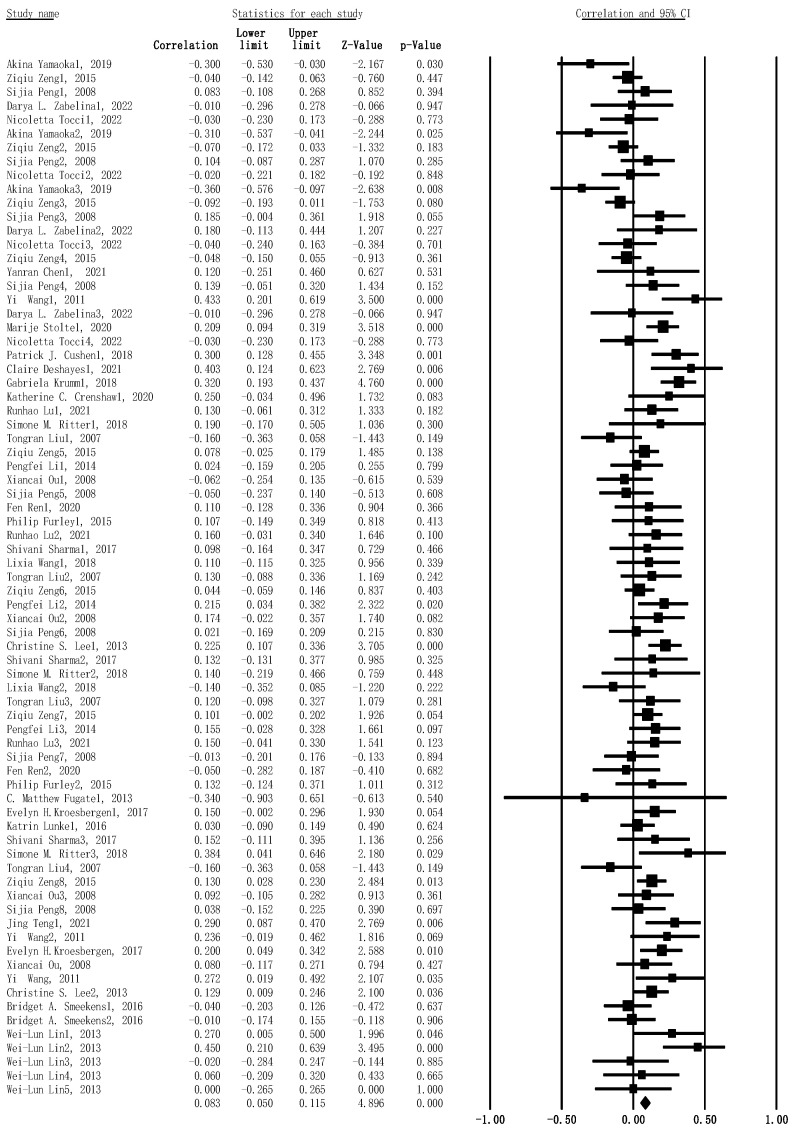
Forest plot for main meta-analysis concerning the association between WMC and creativity. Effect size was presented as z-value in this figure.

As mentioned above, we estimated the pooled effect size by meta-analysis, concerning the r value and the sample size for the included studies. The results demonstrated a significant correlation between WMC and creativity, by pooling these individual effect sizes (*r* = .083, 95% confidence interval (CI): .050–.115, SE = .003, *p* < .001, n = 3104) (see Figure 2).

### 3.2. Subgroup Meta-Analysis

#### 3.2.1. Verbal WMC Tasks and Creativity

**Figure 3:**
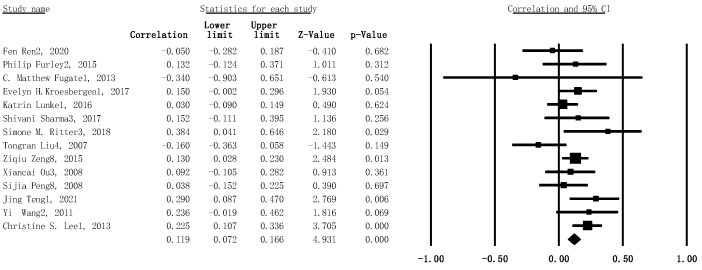
Forest plot with 95% confidence intervals and weights for subgroup meta-analysis, concerning the association between verbal WMC tasks and creativity. Larger positive effect sizes indicate that increased creativity ability is related to verbal WMC.

Likewise, the heterogeneity test was conducted beforehand. Results showed a high heterogeneity for this subgroup meta-analytic model (I^2^ = 39%, *p* = .069). Thus, the meta-analysis, using the random-effects model for investigating the association between verbal WMC and creativity, revealed that the meta-analytic effect for the positive correlation between verbal WMC and creativity reached statistical significance *(r* = .119, 95% CI: .072–.166, SE = .006, *p* < .001, n = 1733) (see Figure 3).

#### 3.2.2. Visual–Spatial WMC Tasks and Creativity

**Figure 4:**
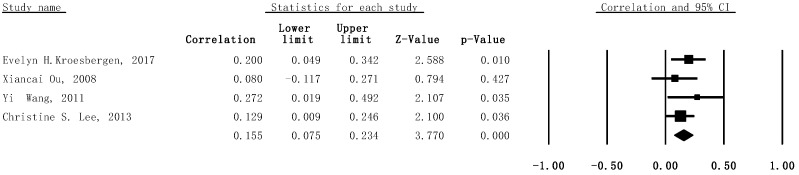
Forest plot with 95% confidence intervals and weights for subgroup meta-analysis, showing the association between visual–spatial WMC tasks and creativity.

Heterogeneity was found to be acceptable for the included studies in the subgroup meta-analytic analysis (I^2^ = 0%, *p* = .58). Thus, the fix-effect model for meta-analysis was built; this demonstrated the statistically significant correlation between WMC and creativity (*r* = .155, 95% CI: .075–.234, SE = .006, *p* < .001, n = 592) (see Figure 4).

#### 3.2.3. Dual-Tasks and Creativity

**Figure 5:**
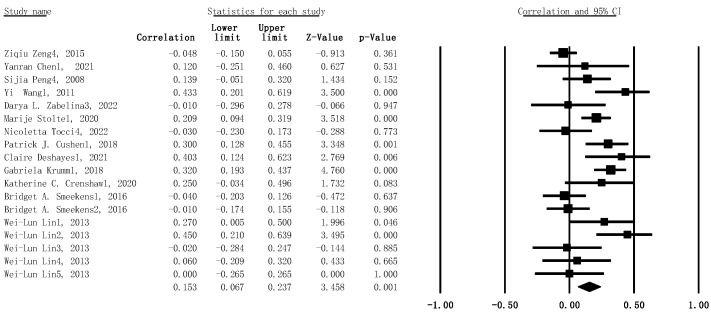
Forest plot with 95% confidence intervals and weights for subgroup meta-analysis, showing the association between dual-tasks and creativity.

Given the high between-study heterogeneity (I^2^ = 70%, *p* < .001), the results of the meta-analysis with the random-effects model showed the statistically significant correlation between dual-task WMC and creativity (*r* = .153, 95% CI: .067–.237, SE = .013, *p* = .001, n = 1602) (see Figure 5).

### 3.3. Moderation-Effect Analysis

Given the high heterogeneity in the meta-analytic model, the moderation-effect analyses were performed to identify factors that may moderate the meta-analytic main effect. Here, the age and cultural contexts of the samples were reported in all the included studies and were modeled as potential moderators separately.

#### 3.3.1. Moderation-Effect of Culture

To examine whether there are confounding factors biasing the meta-analytic effect, we conducted a moderation-effect analysis for cultural contexts (i.e., Western culture and Asian culture) and age groups (i.e., college students (age ≥ 18) and schoolchildren (age < 18)). Results showed the moderating roles of cultural contexts in the meta-analytic effect, with a stronger correlation for participants from Asian cultural backgrounds (*r* = .126, 95% CI: .091–.160, SE = .004, *p* = .000, n = 1282) than that from Western culture (*r* = 0.061, 95% CI: .017–.105, SE = .004, *p* = 0.006, n = 1411); the Q-value was significant (Q = 4.260, *p* = 0.039) (Figure S1).

#### 3.3.2. Moderation-Effect of Age

Null significant findings were observed for the moderating role of age groups in the main meta-analytic effect, including college students (*r* = .078, 95% CI: .048–.109, SE = .005, *p* = .000, n = 1831) and schoolchildren (*r* = .037, 95% CI: −.016–.089, SE = .004, *p* = .168, n = 1080). The Q-value was not significant (Q =1.822, *p* = 0.177) (Figure S2).

#### 3.3.3. Moderation-Effect of WMC Type

Following the moderation analysis of the WMC tasks group, null significant findings were observed for the main meta-analytic effect (Q = 1.360, *p* = 0.507) (Figure S3).

### 3.4. Publication Bias and Quality Assessment

**Figure 6:**
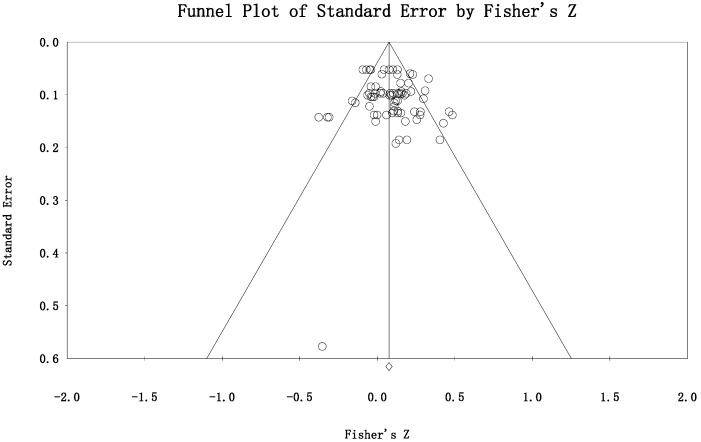
Funnel plot of this study to assess the publication bias. *X*--axis indicates the individual study effect estimates, and *Y*-axis indicates standard errors.

A funnel plot for standard Fisher-Z scores for the included studies can be used to explore the publication bias. The scattered points showed a symmetric distribution (see Figure 6), which indicated no perceived publication bias. To quantify the risk of publication bias, the Egger’s test was conducted. Results showed no prominent publication bias (*e* = .449, *p* = .350). Finally, the evidence quality for the included studies was validated to be acceptable (mean scores for mNOS = 4.65, SD = 0.56, Median = 5).

## 4. Discussion

The main purpose of this study was to clarify the association between WMC and creativity by synthesizing meta-analytic evidence. We found that WMC is significantly positively correlated with creativity by pooling individual effects, indicating that an increased WMC indeed supports human creativity. Furthermore, subgroup meta-analysis was conducted by dividing WMC into three categories, including verbal WMC, visual–spatial WMC and dual-task coordination WMC. The results demonstrated that such associations are robust in different WMC tasks. Lastly, we conducted moderation analysis, which revealed that the correlation between WMC and creativity was moderated by cultural background, with a higher correlation for participants from Asian cultural contexts. On balance, the current study may provide weak evidence to clarify the positive correlation of WMC with creativity. In addition, such associations were found to be robust for the potential impacts of the WMC categories, and the moderating role of cultural background was revealed in this association.

One of the most crucial findings in this study is that there is a statistically significant (but weak) correlation between WMC and creativity. Both theoretical and empirical evidence supports that WMC could positively predict one’s creative ability. As the most important indicator of WM, the WMC is typically described as being the limited capacity for the temporary storage and processing of information (Baddeley 2003). On the basis of controlled attention theory (CAT), creativity is theoretically argued to be a top-down cognitive process that requires considerable cognitive resource control (Beaty and Silvia 2012). As an important component of cognitive control, WM has an imperative ability to upstream regulate complicated creative tasks (Lee and Therriault 2013). In other words, creativity is consistently achieved by extracting relevant knowledge from short-term memory or reconstructing it based on existing knowledge in long-term memory; this needs the substantial support of an adequate WMC. In addition, the positive association between WMC and creativity has been validated in a recent large-sample neuroimaging study (Takeuchi et al. 2020); this revealed the overlapping co-activated areas for the WM task and creativity task. Thus, it may provide robust evidence to clarify the positive association of WMC with creativity; this offers insight into addressing this long-lasting debate.

By using subgroup analysis, this study also found that the positive correlations between WMC and creativity were consistent, irrespective of the WMC category (i.e., verbal WMC, visual–spatial WMC, and dual-task coordination WMC). This finding attempted to answer whether the conflicting results, derived from previous studies, were attributed to a heterogenous WMC category. Supporting this, measuring WMC performance was argued to be comparable across different sensory pathways (e.g., visual and verbal) (Xu et al. 2017). In addition, Lee and colleagues (2011) well documented an increased activation in the related brain regions (medial temporal lobe, MTL) when working memory demand was increased, regardless of the type of stimulus (e.g., visual and auditory) (Lee and Rudebeck 2010). This evidence may lead us to draw the conclusion that the positive association between WMC and creativity is robust, or more boldly, to infer that the existing conflicting results may not be ascribed to sensory processing in WMC tasks.

To clarify the impact of potential confounding factors on the meta-analytic effects, moderation analyses were drawn for the cultural background of participants (i.e., Western culture and Asian culture) and age groups (i.e., adolescents and adults), respectively. Interestingly, the meta-analytic effect for the association between WMC and creativity was significantly moderated by cultural background. Specifically, compared to participants with Western cultural background, participants in the Asian cultural environment reported a stronger correlation between WMC and creativity. This finding could be explained partly by the relativity of the creativity theory (Guilford 1950). This theoretical framework elucidated the fact that creativity could be defined and evaluated specifically in different cultural environments, due to a lack of a practical criterion for creativity, with liberal scopes in western cultures (e.g., arts that required less deliberative cognitive process) (Hempel and Sue-Chan 2010). Conversely, measuring creativity in Asian cultures required strict executive functions (especially in WM) in creative tasks, such as problem-solving, deliberative reasoning and insight inference (Leung and Wang 2015). Rudowicz (2003) argued that the influence of culture on creativity is complex and highly interactive, involving historical, social, and personal cross-cultural factors (Rudowicz 2003). The key to the cross-cultural study of creativity is uncovering whether the definition and operationalizations of creativity from one culture can be validly applied to another one; this includes the eastern–western cultural gap or the conservative/traditional-liberal cultural gap. To provide evidence for this, some studies, comparing creativity between Westerners and Asians, demonstrated that the performance of creative activities was higher in people from cultural environments that highlighted creativity values (Niu and Sternberg 2002, 2003). Furthermore, one interesting finding was that Western cultures valued individualistic, intuitive, and artistic processing in creative activities, while Asian culture stressed collectivistic, cognitive, and deliberative thoughts (Goncalo and Staw 2006). That is to say, the gap between cultures, and their required involvement of cognitive processing (i.e., WM) in creative tasks, may be a crucial factor biasing the association between WM and creativity. This study indicates that the cultural gap between participants may be a source of conflict, caused by the results of existing studies.

## 5. Limitation

Although this study clarifies the association between WMC and creativity, several limitations should be acknowledged. Due to the strict inclusion and exclusion criteria, the total sample size (n < 3500) and evidence (study, k = 28) seem to be inadequate. Therefore, the nuance of these variations in task types of WM or creativity cannot be examined currently. Thus, future studies are needed to provide neuroimaging evidence to further confirm the association between them. Additionally, extending the main conclusion of the current study is prudent, because the total effect size for such an association is relatively small (though reaching statistical significance).

## 6. Conclusions

This study provides evidence to clarify the statistically significant positive association between WMC and creativity, though it has a weak strength. Further, the present study revealed that such associations exist across different types of WMC measurement (i.e., verbal WMC task, visual–spatial WMC task and dual WMC task), indicating that the conflicting results for the association between them are not biased by measure heterogeneity. This study also demonstrates that the cultural gap may confound the association between WMC and creativity.

## Figures and Tables

**Table 1:** Summary of characteristics of included studies. N.A. = not applicable.

Author	Publication Time	Region	Sample Size	Age	Gender		Subject Category	Working Memory Measures	Creativity Measures	Variable Relation
					Male	Female				
(Yamaoka and Yukawa 2020)	2019	Japan	52	18.96 ± 1.21	19	33	College students	OSPAN and SSPAN	UUT	Dual-tasks and creativity
(Zeng 2015)	2015	CN	364	9.4 ± 0.45	182	182	Schoolchildren	Digit Span Backwards/N-Back	CAT, TTCT	Verbal WMC tasks/Dual-tasks and creativity
(Peng 2008)	2008	CN	108	17.66 ± 0.74	62	46	Students	Digital comparison task/space tracking task	Creative Thinking Test	Verbal WMC tasks/Visual–spatial and creativity
(Zabelina et al. 2019)	1 February 2022	USA	47	29.26 ± 7.93	23	24	Neuro-typical adult	WMC updating tasks	ATTA, CAQ	Dual-tasks and creativity
(Tocci et al. 2022)	10 March 2022	Italy	95	7.8 ± 1.3	47	48	Schoolchildren	Random Number Generation Task.	TTCT	Dual-tasks and creativity
(Chen 2021)	2021	CN	30	NA	NA	NA	Students	Short-term memory task	AUT	Dual-tasks and creativity
(Wang 2011)	2011	CN	60	18–24	NA	NA	College students	Operation word breadth task/point matrix task	WCAP	Verbal WMC tasks/Visual–spatial WMC tasks/Dual-tasks and creativity
(Stolte et al. 2020)	2 June 2020	The Netherlands	278	9.71 ± 0.93	139	139	Schoolchildren	the Monkey Game/the lion game	the Mathematical Creativity task	Dual-tasks and creativity
(Cushen and Wiley 2018)	2 August2018	USA	120	19.39 ± 1.74	NA	NA	College students	OSPAN and SSPAN	RAT	Dual-tasks and creativity
(Deshayes et al. 2021)	7 May 2021	Germany	45	14.13 ± 3.25	22	23	Normal person	WMC updating tasks	TTCT	Dual-tasks and creativity
(Krumm et al. 2018)	30 July 2018	Argentina	209	NA	NA	NA	Schoolchildren	WISC-IV	TTCT	Dual-tasks and creativity
(Crenshaw and Miller 2022)	9 May 2020	USA	49	9±0.25	19	30	Schoolchildren	WAIS –III	AUT	Dual-tasks and creativity
(Lu et al. 2021)	14 April 2021	CN	107, 68, 64	20.45 ± 3.31, 21.49 ± 2.26, 23.23 ± 3.83	45, 31, 27	62, 37, 37	College students	Verbal tasks and Visual–spatial task/The tapping task	TTCT	Verbal WMC tasks/Visual–spatial WMC tasks and Verbal\Figure creativity
(Ritter et al. 2018)	31 July 2018	The Netherlands	32	19.7	NA	NA	College students	DS	RAT	Verbal WMC tasks and creativity
(Liu and Shi 2007)	2007	CN	83	9/10/11 ± 0.25	50	33	Schoolchildren	Sternberg WM Paradigm	WCAP, CAT	Verbal WMC tasks and creativity
(Li 2008)	2014	CN	116	18–24	31	85	College students	OSPAN	UUT	Verbal WMC tasks and creativity
(Xiancai Ou 2008)	2008	CN	101	19.62	65	44	College students	Operation—word width task/point matrix space width task	Creative Thinking Scale	Verbal WMC tasks/Visual–spatial WMC tasks and creativity
(Ren et al. 2020)	2020	CN	70	19.84 ± 1.46	27	43	College students	Verbal WM tasks	WPMF	Verbal WMC tasks and creativity
(Furley and Memmert 2015)	10 February 2015	Germany	61	23.48 ± 3.6	61	0	Soccer athletes	OSPAN	DT test	Verbal WMC tasks and creativity
(Sharma and Babu 2017)	2 February 2017	India	58	52.05, 57.21, 62.05	25	33	Middle-aged and older adults	PGI memory scale	TTCT	Verbal WMC tasks and creativity
(Wang et al. 2018)	21 September 2018	CN	78	21.54 ± 1.33, 21.73 ± 1.45	9	69	Neuro-typical adult	OSPAN, RAPM, number-letter task	AUT	Verbal WMC tasks/Dual-tasks and creativity
(Lee and Therriault 2013)	5 June 2013	USA	265	20.33 ± 2.54	59	206	College students	Symmetry Span task/Backward Digit Span task	AF tasks, DT tests, CT tests	Verbal WMC tasks/Visual–spatial tasks and creativity
(Fugate et al. 2013)	30 August 2013	USA	6	NA	NA	NA	Gifted Students Without ADHD characteristics	The Woodcock Johnson III	TTCT	Verbal WMC tasks and creativity
(Kroesbergen and Schoevers 2017)	22 December 2017	The Netherlands	166	9.66 ± 0.58	79	87	Schoolchildren	Two computerized WM tasks	TCT-DP, MCT	Verbal WMC tasks/Visual–spatial and creativity/mathematical creativity
(Lunke and Meier 2016)	28 July 2016	Switzerland	270	26.19 ± 8.52	NA	NA	Neuro-typical adult	RST	ACDC	Verbal WMC tasks and artistic Creativity
(Teng 2021)	2021	CN	89	21.96	19	70	College students	OSPAN	AUT, TTCT, Creative tasks	Verbal WMC tasks and Artistic Creativity
(Smeekens and Kane 2016)	7 May 2013	CN	55	20.3 ± 1.2	21	34	College students	N-back tasks	DT tasks	Dual-tasks and creativity
(Lin and Lien 2013)	15 February 2016	USA	173, 142	NA	NA	NA	College students	OSPAN and SSPAN	AUT, SART	Dual-tasks and creativity

Notes: TTCT: Torrance Tests of Creative Thinking, TCT-DP: Test of Creative Thinking-Drawing Production, ATTA: Abbreviated Torrance Test for Adults, DT: Divergent thinking tests, CT: Convergent thinking tests, WPMF: Williams Prefer Measurement Forms, WCAP: Williams Creativity Assessment Packet, UUT: Unusual uses task, AUT: Alternative Uses Task, CAT: Consensual Assessment Technique, CAQ: Creative Achievement Questionnaire, AF: Associative fluency tasks, RAT: Remote Association Test. ACDC: The Artistic Creativity Domains Compendium. Verbal WMC task: test phonological loop (Word recall forwards, Digit Recall, Non-word List Recall, Word List Recall, OSPAN; visual–spatial WMC task: test visual–spatial sketchpad (Corsi block task, Dot matrix, matrix pattern, spatial span, Block Recall, SSPAN; dual WMC task: including both dual verbal and visual–spatial WMC tasks or complex WMC task (N-back task, recall tasks, tasks including both verbal and visual–spatial WMC tasks. WISC: Wechsler Intelligence Scale for Children–Fourth Edition, WAIS: Wechsler Adult Intelligence Scale.

## Data Availability

All the materials regarding this study have been deposited in Open Science Framework (OSF): https://osf.io/3g2j9/ (accessed on 1 January 2023).

## Supplementary Materials

The following supporting information can be downloaded at: https://www.mdpi.com/article/10.3390/jintelligence11010015/s1, Figure S1: The results of the moderation-effect of culture; Figure S2, The results of the moderation-effect of age; Figure S3, The results of the moderation-effect of WMC types group.
