# Supplementary material for: A Positive Association between Working Memory Capacity and Human Creativity: A Meta-Analytic Evidence
Source: J Intell. 2023 Jan 13;11(1):15. doi: 10.3390/jintelligence11010015 (PMC9861316; doi:10.3390/jintelligence11010015)
Supplement: Supplementary file 1 [file jintelligence-11-00015-s001.zip › jintelligence-1970926-supplementary.pdf]

Supplementary Materials for

Increased Working Memory Capacity Supports Human Creativity: A Meta-analytic Evidence

Contents

3 of 3 Supplementary Figures

Groups		Effect size and 95% interval			Test of null (2-Tail)		Heterogeneity				Tau-squared			
Group	Number Studies	Point estimate	Lower limit	Upper limit	Z-value	P-value	Q-value	df (Q)	P-value	I-squared	Tau Squared	Standard Error	Variance	Tau
**Fixed effect analysis**														
Asian	30	0.126	0.091	0.160	7.071	0.000	43.020	29	0.045	32.589	0.005	0.004	0.000	0.069
Western	20	0.061	0.017	0.105	2.729	0.006	22.480	19	0.261	15.480	0.002	0.004	0.000	0.044
Total within							65.500	48	0.047					
Total between							5.181	1	0.023					
Overall	50	0.101	0.073	0.128	7.230	0.000	70.680	49	0.023	30.674	0.004	0.003	0.000	0.066
**Mixed effects analysis**														
Asian	30	0.131	0.086	0.175	5.707	0.000								
Western	20	0.061	0.011	0.110	2.389	0.017								
Total between							4.260	1	0.039					
Overall	50	0.099	0.066	0.132	5.832	0.000								

Figure S1. The results of the moderation-effect of culture.

Groups		Effect size and 95% interval			Test of null (2-Tail)		Heterogeneity				Tau-squared			
Group	Number Studies	Point estimate	Lower limit	Upper limit	Z-value	P-value	Q-value	df (Q)	P-value	I-squared	Tau Squared	Standard Error	Variance	Tau
**Fixed effect analysis**														
college student	48	0.078	0.048	0.109	5.022	0.000	85.988	47	0.000	45.341	0.010	0.005	0.000	0.099
school-children	20	0.033	0.002	0.063	2.083	0.037	48.517	19	0.000	60.838	0.008	0.004	0.000	0.088
Total within							134.505	66	0.000					
Total between							4.321	1	0.038					
Overall	68	0.055	0.034	0.077	5.023	0.000	138.826	67	0.000	51.738	0.009	0.003	0.000	0.095
**Mixed effects analysis**														
college student	48	0.083	0.041	0.126	3.827	0.000								
school-children	20	0.037	-0.016	0.089	1.380	0.168								
Total between							1.822	1	0.177					
Overall	68	0.065	0.032	0.098	3.838	0.000								

Figure S2. The results of the moderation-effect of age.

Groups		Effect size and 95% interval			Test of null (2-Tail)		Heterogeneity				Tau-squared			
Group	Number Studies	Point estimate	Lower limit	Upper limit	Z-value	P-value	Q-value	df (Q)	P-value	I-squared	Tau Squared	Standard Error	Variance	Tau
**Fixed effect analysis**														
dual	18	0.131	0.086	0.175	5.746	0.000	56.157	17	0.000	69.728	0.023	0.013	0.000	0.150
verbal	13	0.100	0.048	0.151	3.778	0.000	17.505	12	0.132	31.448	0.004	0.006	0.000	0.067
visual spatial	4	0.155	0.075	0.234	3.770	0.000	1.963	3	0.580	0.000	0.000	0.006	0.000	0.000
Total within							75.625	32	0.000					
Total between							1.548	2	0.461					
Overall	35	0.123	0.092	0.154	7.743	0.000	77.173	34	0.000	55.943	0.012	0.005	0.000	0.108
**Mixed effects analysis**														
dual	18	0.153	0.067	0.237	3.458	0.001								
verbal	13	0.101	0.034	0.168	2.936	0.003								
visual spatial	4	0.155	0.075	0.234	3.770	0.000								
Total between							1.360	2	0.507					
Overall	35	0.131	0.087	0.175	5.782	0.000								

Figure S3. The results of the moderation-effect of WMC types group.
